# Effects of before-school physical activity programs on preschool and school children: a systematic review

**DOI:** 10.3389/fpubh.2026.1784551

**Published:** 2026-07-28

**Authors:** Miren Garin-Atorrasagasti, Markel Rico-González, Boryi A. Becerra-Patiño, José Francisco López-Gil, Jorge Olivares-Arancibia

**Affiliations:** 1 Department of Didactics of Musical, Plastic and Corporal Expression, University of the Basque Country, EHU, Leioa, Spain; 2 Management and Pedagogy of PA and Sport (GPAFD), Faculty of Physical Education, National Pedagogical University, Bogotá, Colombia; 3 School of Medicine, Universidad Espíritu Santo, Samborondón, Ecuador; 4 Faculty of Health Sciences, Universidad Autónoma de Chile, Santiago, Chile; 5 AFySE Group, Faculty of Education, Research in Physical Activity and School Health, School of Physical Education, Universidad de las Américas, Santiago, Chile

**Keywords:** children, exercise, health, physical education, school, sports, systematic review

## Abstract

**Introduction:**

Given that several preschools and schoolchildren do not meet physical activity (PA) guidelines, the interest in including short activity breaks during school hours has become a popular trend among professionals in educational settings. Among them, before-school PA breaks have been highlighted as a suitable alternative. This systematic review aims to analyze the effects of before-school PA breaks on preschool-aged and school-aged children.

**Material and methods:**

Following Preferred Reporting Items for Systematic Reviews and Meta-analyses (PRISMA) guidelines, five databases were searched for articles published before September 11, 2025. The PICOS framework (population; intervention; control; outcomes and study design) targeted children up to 12 years of age who had participated in before-school PA programs. Study quality was evaluated using different scales depending on the type of study.

**Results:**

Of the 526 studies identified, 16 met the inclusion criteria, encompassing 2,361 children. PA intervention before class can improve physical fitness and academic performance. However, some variables, such as attention and concentration; physical characteristics, such as BMI; and social aspects, such as quality of life and physical and verbal school bullying, depend on how much physical improvement children achieve.

**Discussion:**

This review suggests incorporating before-school-based interventions that consider PA performance can constitute an important strategy for children's development and growth.

**Systematic review registration:**

PROSPERO CRD420251240838.

## 1 Introduction

Physical activity (PA) is essential for promoting children's health and development (1). In fact, PA has been shown to have positive effects on physiological, cognitive, and socio-emotional wellbeing (2). Although these benefits are widely known, PA levels among the world's youngest population are lower than they should be (3), given that some children and adolescents do not meet the recommended level of PA suggested for health benefits (< 11.26%) (4). In fact, 81% of children do not engage in PA (5), and preschool-aged children spend 69% of their time at school engaged in sedentary activities (6). Therefore, changing this situation has become a challenge for professionals working with children. Addressing this challenge requires a comprehensive understanding of the multiple factors that influence children's PA behavior, especially since this is not merely a health issue but a social one in which educational institutions and families are responsible for providing the necessary conditions to achieve these recommended levels through direct practice and mutual cooperation.

To understand the multifaceted effects of PA on children, theoretical frameworks are needed that address both individual motivational processes and broader environmental influences. Various theories exist, including self-determination theory and the socio-ecological model, among others. Self-determination theory posits that participation in PA is enhanced when interventions meet basic psychological needs related to autonomy, competence, and relatedness, thereby promoting intrinsic motivation and sustained participation (7). Complementarily, the socio-ecological model posits that children's PA behavior is determined by multiple interacting levels, including individual characteristics, interpersonal relationships, organizational environments, community environments, and normative contexts (8).

Given this growing trend toward a sedentary lifestyle starting in childhood and the negative consequences this could have at this stage, PA habits are fundamental to the development of future lifestyles and health (9). Among other factors related to this lack of mobility, various studies have revealed a high prevalence of childhood obesity, which increases the risk of developing chronic diseases in adulthood (10). This rise in obesity, along with the accumulation of excess fat in children, leads to the development of various conditions, including diabetes, cardiovascular problems, various types of cancer, and mobility issues (11). On the other hand, engaging in PA improves self-esteem and body image, factors linked to mental health conditions that are currently becoming a global pandemic (12). Furthermore, as a social activity, it can lead to a reduction in gender, social, religious, and racial inequalities (13), as well as improve other social skills, such as emotional regulation (14). Finally, if PA is practiced regularly, it can improve cognitive performance, particularly attention and cognitive function (15–17).

Therefore, although the World Health Organization (18, 19) provides recommendations on PA to improve the physical health of children and adolescents (4), the amount of time children over the age of 6 spend on PA is gradually decreasing (20). As a result, sedentary habits are beginning to increase, mainly due to excessive use of all types of screens, such as television, smartphones, and computers (11, 21). In this context, given the amount of time children spend in school, education plays a crucial role in promoting healthy habits, especially during the early years of life (22). Furthermore, even though the PA children engage in outside of school hours may not be sufficient to meet the recommendations (9), school becomes an ideal opportunity to promote and increase PA participation (23).

Within this framework, schools emerge as key institutional settings where structured PA interventions can address multiple ecological levels simultaneously, making them strategic environments for promoting healthy behaviors among preschool- and school-aged populations (24). These theoretical perspectives are particularly relevant when examining the consequences of insufficient PA in childhood. According to available scientific evidence, preschool programs have been shown to promote health and wellbeing, particularly by improving readiness for learning (25). One existing program, called Build Our Kids' Success (BOKS), has reported benefits from its implementation in physical and mental health, as well as in the academic and cognitive performance of elementary school students (25). Meanwhile, another program called Active-Start reported that the implementation of such programs produces significant improvements in emotional wellbeing, mood, and readiness for learning (26). From this perspective, there is a concern to implement various pre-school programs to encourage greater participation in PA; therefore, such methodologies should be incorporated into policies and guidelines on PA, as well as into professional development initiatives to support these efforts (27, 28).

Among these new methodologies, active breaks (ABs) are suitable strategies for enhancing children's PAs. ABs are short, healthy PA activities that are implemented during school hours and within the classroom (29). These methodologies, created as alternatives to respond to sedentarism and a lack of PA (30), with the aim of bringing movement to classrooms, have gained increasing strength (31). The importance of this methodology can be reinforced via principal component analysis, which highlights AB as an important factor that can affect healthy habits (32).

In this sense, various studies have shown that implementing AB programs in the classroom has significant benefits for both students and teachers. These methodological interventions have improved selective attention and concentration (30, 33). Furthermore, after their application, there is greater readiness for work and an increase in the learning atmosphere in the classroom (30, 34). In this context, AB is associated with increased motivation, cognitive and academic performance, and more efficient use of school time (35, 36). In the systematic review conducted by Melguizo-Ibáñez et al. (37), which focused on AB to improve attention, several variables were analyzed, such as the time of application, the benefits obtained and the age groups, but the time of day at which these interventions are carried out was not specifically addressed.

This deficiency is particularly important since preschool PA programs can offer distinct benefits in terms of activation, attention, and attitudes toward learning. Furthermore, there remains a lack of scientific literature evaluating the impact of before-school PA programs and/or interventions on children up to 12 years of age, even though this period is crucial for the development of motor and cognitive skills. This deficiency is particularly important since preschool PA programs can offer distinct benefits in terms of activation, attention, and attitudes toward learning. Furthermore, the scientific literature remains limited for individuals aged 3–6 years, even though this period is crucial for the development of motor and cognitive skills. To the best of our knowledge, no systematic review has been reported that analyzes the effects of active breaks before school hours in early childhood education settings. Therefore, it is necessary to gain a detailed understanding of the impact of active breaks before school hours in early childhood and elementary school settings, the conditions under which they are implemented, and the potential health implications of their early implementation. For this reason, the objective of this systematic review was to analyze the effects of pre-school physical activity breaks on preschool-aged and school-aged children.

## 2 Materials and methods

### 2.1 Experimental approach to the problem

The preferred reporting items for systematic reviews and meta-analyses (PRISMA) criteria (38) and guidelines for conducting systematic reviews in sport sciences (39) were followed to conduct this systematic review. The International Prospective Register of Systematic Reviews (PROSPERO) is number CRD420251240838.

### 2.2 Information sources

A systematic search of five main databases (PubMed, ProQuest, Scopus, Web of Sciences, and SPORTDiscus) was performed to identify articles published prior to September 11, 2025. All the databases were checked the same day.

### 2.3 Search strategy

The search was explicitly stated via the PICOS (Patient, Problem, or Population—Intervention—Comparison, Control, or Comparator—Outcome[s]) design. The same search strategy was used to look for articles in each of the databases. The search strategy in all of the databases was used using the filter “Title/Abstract,” but in Scopus the filter was “Title, Abstract, and Keywords,” because only “Title/Abstract” was not possible. No other filter was applied (field, date restriction, language, or article type). Journal names and authors of manuscripts were not hidden from the author. After each article was downloaded, each one was examined for eligibility using the inclusion-exclusion criteria one at a time. An article was downloaded and included in the review, and its data were exported to Table 1 if it satisfied all the inclusion criteria. An article was removed with a comprehensive explanation if it did not satisfy all the inclusion requirements. The titles and abstracts of the publications contained the following search words (see Table 2):

**Table 1:** Characteristics of included studies.

References	Sample	Before-school program	Test/instruments	Variables	Results	Conclusions
Koncz et al. (43)	Hungary, 51 preschoolers (mean 6.8 y)	Mindfulness-relaxation, 1 week, 5x 30 min	Digit span, Go/No-Go, cortisol sampling	Executive functions, cortisol	No EF effect: boys showed reduced cortisol postschool entry	Short mindfulness feasible; stress-buffer in boys
Stylianou et al. (44)	USA, 88 students, grades 3–4	Running/walking club; 8 weeks, 3x/week, 20 min	Accelerometers	Steps, MVPA	Significant increase in steps and MVPA; no compensatory decrease	Before-school walking/running clubs effectively increase PA
Kulp and Zhu (45)	USA, 84 children, grades 4–5	10 weeks, 1x/week, 45 min aerobic program	Fitness tests, reading/math assessments	Fitness, academic performance	Improved fitness and reading scores; no effect on math	Weekly before-school PA benefits fitness and reading
Hoza et al. (46)	USA, 202 children, mean age 6.8 y	Aerobic activity vs. sedentary; 12 weeks, 31 min/day	DBD rating scale, teacher reports	ADHD symptoms, emotion, peer functioning	Reduced inattention and mood problems; improved behavior	Before-school PA reduces ADHD-related symptoms
Smith et al. (47)	USA, 17 children (K−3), ADHD risk	26 min/day, 8 weeks, MVPA in stations	DBD, BOT-2, shape school, teacher/parent ratings	ADHD symptoms, motor, cognition, behavior	Improvements in inhibition, behavior, ADHD symptoms	Before-school PA promising for ADHD symptom reduction
Cradock et al. (48)	USA, 426 students, grades K−6	BOKS; 12 weeks, 3x/week, 40 min	Accelerometers	Steps, MVPA, VPA	Higher PA on program days	BOKS increases children's PA before school
García-Hermoso et al. (49)	170 schoolchildren, 4th grade (8–10 years), 3 low-SES public schools, Santiago, Chile	Active-Start; before-school program of cooperative physical games at moderate-to-vigorous intensity, 5x/week, 8 weeks (~39 sessions), 8:00–8:30 a.m. Also included adapted sports games, playground games, dance, and social cool-down activities	BP measured with validated oscillometric monitor (Omron HEM 705 CP; lowest of two readings after rest); MAP calculated from SBP/DBP. Physical activity and sedentary time assessed via wrist-worn GENEActiv accelerometer (7 days), processed in R using the GGIR package. Anthropometry (weight, height, BMI) measured with standard equipment; maturity status estimated via PHV	SBP, DBP, MAP; BP category (normal < 90th percentile vs. elevated/hypertension ≥90th percentile); sedentary time (moderator, min/day); MVPA; BMI, PHV, sex, age, school (covariates)	Active-Start produced significant within-group reductions in SBP, DBP, and MAP (all *p* < 0.01, small-to-moderate effect sizes), but these changes were not significantly different from the control group (*p* > 0.05). The intervention's effect on SBP and MAP was moderated by sedentary time: benefits were only significant below ~657–659 min/day of sedentary time; above this threshold, the effect disappeared. More sedentary children also had lower MVPA	Sedentary time moderates exercise-related BP benefits in children — the intervention's effect is lost in highly sedentary children regardless of MVPA. Reducing sedentary behavior, not just increasing activity, appears key to cardiovascular benefit
Terson De Paleville and Immekus (50)	USA, 60 elementary students, 8–9 y	Minds in Motion & Yoga; 6 weeks, 3x/week, 25 min	BOT-2, Woodcock-Johnson Tests	Motor proficiency, reading, math	Improved motor skills and academic measures in intervention groups	Short PA/yoga before school enhances motor and academic skills
Goh et al. (51)	USA, 138 children, mean 10 y	6 weeks, 3x/week, SEL-based PA	DESSA-Mini	Social-emotional learning	Improved SEL competencies	PA integrated with SEL before school supports wellbeing
Hormazábal-Aguayo et al. (52)	146 4th-graders (8–10 y) from 3 vulnerable public schools, Santiago, Chile (Intervention *n* = 88; Control *n* = 58)	Active-Start: 8-week, 5x/week, before-school cooperative/recreational games (moderate-vigorous intensity) promoting fair play, rules, and conflict resolution. Both groups kept regular PE	Bullying: CUBE questionnaire, Anthropometry: BMI, Maturity: PHV, PA: GENEActiv accelerometer	Physical Verbal, and social-exclusion bullying, MVPA	Significant reductions in physical and verbal bullying victimization post-intervention; no effect on social exclusion bullying	An 8-week before-school PA program reduced physical and verbal bullying in disadvantaged children; low-cost and potentially scalable to improve school climate, though limited by small school sample and low generalizability
Whooten et al. (53)	USA, 707 students, 24 schools	BOKS; 12 weeks, 2–3x/week	BMI, wellbeing surveys	BMI z score, socioemotional wellbeing	BMI z score improved (3x/week group), wellbeing increased	Frequent BOKS sessions improve health indicators
García-Hermoso et al. (54)	Chile; *n* = 170 children (8–10 years old; 45.8% and 41.5% girls in control and intervention groups, respectively) from 3 public schools, low SES	Active-start, 8 weeks, 5 days/week, 30 min/session (cooperative physical games, dance, playground activities, MVPA intensity) delivered at 8:00–8:30 am before classes	d2 Test of Attention (paper-pencil); official school grades in language & math; ALPHA test battery: JAMAR dynamometer (Hydraulic Hand Dynamometer^®^ Model PC-5030 J1), standing long jump, 4 × 10 m shuttle-run, 20 m shuttle-run (VO_2_max by Léger equation); TANITA SC-331 bioelectrical impedance analyzer; Seca 769 scale & Seca 220 stadiometer for BMI; waist circumference tape; GENEActiv accelerometer	Selective attention, concentration, language, math, BMI, waist circumference, fat mass, fat-free mass, muscular fitness, motor ability, CRF	Improvements in language and math performance. Reduction in fat mass and increase in fat-free mass	An 8-week before-school PA program improved academic performance, body composition, muscular and cardiorespiratory fitness. CRF moderated effects on attention and concentration; benefits were evident only in children with greater CRF gains
Brackney et al. (55)	USA, 200 children	Dance-based play sessions	Heart rate, PA logs	PA intensity, enjoyment	Children engaged in MVPA; enjoyed activities	Dance-based PA feasible to promote MVPA
Tompkins et al. (56)	USA, 42 students	Self-selected PA sessions	Heart rate monitors, RPE	HR, perceived exertion	Obese children had higher HR and RPE vs. normal weight	Self-paced PA feasible; differences by weight status
Willis et al. (57)	USA, 88 students, 4th−5th grade	8 weeks, 3x/week, 15–20 min; cooperative PA, TPSR model	Steps, Stroop test, academic progress	On/off-task behavior, PA, academics	Increased PA, SEL; no significant academic improvement	Before-school RTI improves behavior and PA
Korcz et al. (58)	Poland, 88 children, 8–9 y	Active before school bell; 12 weeks, 3x/week, 15 min (PA vs cognitive)	Eurofit, academic skills battery, PA questionnaires	Academic skills, fitness, PA attitudes	PA group improved visual-spatial; both experimental > control in language skills	PA before school effective for academic and fitness outcomes

DBD, disruptive behavior disorders rating scale; BOT-2, Bruininks–Oseretsky Test of motor proficiency, 2nd ed; WRAML-2, wide range assessment of memory and learning, 2nd ed.; ActiGraph GT3X, tri-axial accelerometer for PA assessment; PACER, progressive aerobic cardiovascular endurance run (fitness gram); MVPA, moderate-to-vigorous physical activity; VPA, vigorous physical activity; RTI, response to intervention; SEL, social and emotional learning; HBSC, health behavior in school-aged children survey; APAS, attitudes toward PA scale; DBP, diastolic blood pressure; SBP, systolic blood pressure; MAP, mean arterial pressure; BP, blood pressure; RCT, randomized controlled trial; BMI, body mass index; PHV, peak height velocity; SES, socioeconomic status.

**Table 2:** Inclusion and exclusion criteria for study selection.

No.	Item	Inclusion criteria	Exclusion criteria	Search coherence
1	Population	Children attending preschools or schools (until 12 years old)	Children with more than 12 years old (from middle school or high school) Children out of school (ambulatory children, children with medical treatment)	(Preschool OR Kindergarten OR “early childhood” OR “primary education” OR “elementary education” OR school)
2	Intervention/exposure	Children participating in a before school PA program	Children not participating in a before-school Children participating in a school program not only before-school Staff experiences Studies based on surveys Study protocols	(“before school”) AND (“physical activity” OR “physical education” OR exercise OR movement OR activity OR sport OR fitness OR aerobic OR training OR performance) AND (program^*^ OR intervention)
3	Comparison	–	–	–
4	Outcome(s)	Any effect derived from before-school program and extracted directly from children	Outcomes not related to effects (e.g. distance covered) Parents or caregivers perceptions	
5	Study design	–	–	–
6	Other criteria	Peer-reviewed, original, full-text studies	Non-peer-reviewed, non-original	–

(Preschool OR Kindergarten OR “early childhood” OR “primary education” OR “elementary education” OR school) AND (“before school”) AND (“physical activity” OR “physical education” OR exercise OR movement OR activity OR sport OR fitness OR aerobic OR training OR performance) AND (program^*^ *OR intervention)*.

### 2.4 Eligibility criteria

One author extracted the data (title, authors, date, and database) from the papers and entered it into an Excel spreadsheet (Microsoft Corporation, Redmond, WA, USA) to eliminate duplicates. To choose the studies that satisfied all the inclusion criteria, the remaining articles were evaluated by two authors (Table 2). A third author should decide whether to include or exclude an article if these two authors cannot agree.

### 2.5 Data extraction

An Excel spreadsheet was used to prepare the data extraction in compliance with the Cochrane Consumers and Communication Review Group's data extraction template. All of the chosen studies' inclusion and exclusion criteria were evaluated via a spreadsheet. Reasons for the exclusion of full-text publications from the analysis were noted. The spreadsheet contained all of the records. Data extraction was performed by two authors independently. Any discrepancies were resolved through discussion, and when a consensus could not be reached, a third author acted as an arbitrator. The agreement between the authors was *k* = 0.91.

Once all records were selected and downloaded, the following information was extracted: sample, intervention, test/instruments, variables, results, and conclusions.

### 2.6 Quality of the studies

Two review authors (J.O.-A and B.A.B.-P) independently assessed the risk of bias of each study using the RoB 1 criteria described in the Cochrane Handbook for Systematic Reviews of Interventions (40). Any disagreement in the risk of bias assessment was resolved by consensus or by involving another review author. We assessed the risk of bias according to the following domain: random sequence generation. Allocation concealment. Blinding of participants and personnel. Blinding of outcome assessment. Incomplete outcome data. Selective outcome reporting. Other sources of bias (e.g., potential sources of bias reported by the authors, participant selection, study design). For nonrandomized trials, we used the risk of bias in nonrandomized studies of interventions (ROBINS-I). We judged the risk of bias in each domain and the overall risk of bias for each outcome in each study on the basis of the ROBINS-I guidelines (41). We used the following overall risk of bias criteria. Low risk of bias: the study was judged to be at low risk of bias for all domains. Moderate risk of bias: the study was judged to be at low or moderate risk of bias for all domains. Serious risk of bias: the study is judged to be at serious risk of bias in at least one domain but not at critical risk of bias in any domain. Critical risk of bias: the study is judged to be at critical risk of bias in at least one domain. No information: There is no clear indication that the study is at serious or critical risk of bias and that there is a lack of information in one or more key domains of bias. To assess the risk of bias in retrospective studies, we used the “JBI Critical Appraisal Checklist for Case–Control Studies.” This tool was originally designed for retrospective case–control studies, so we adapted it accordingly. We did not consider Domains 2 and 3, as the included studies did not involve control groups. Finally, for the case series study, we used the “JBI Critical Appraisal Checklist for Case Series” (42).

## 3 Results

### 3.1 Identification and selection of studies

**Figure 1:**
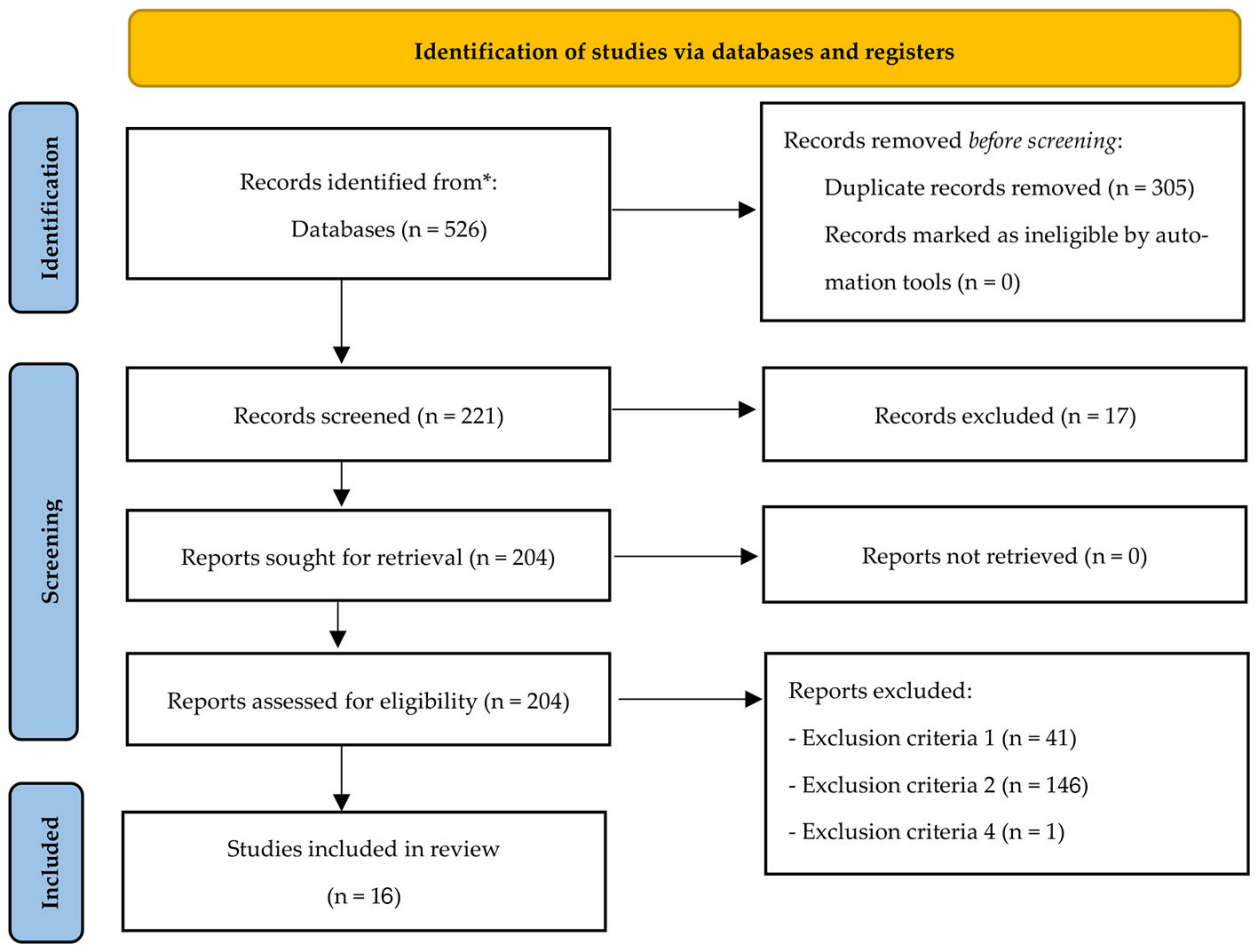
Flow diagram of the study.

A total of 526 original articles were retrieved [PubMed (*n* = 75), ProQuest (*n* = 143), SCOPUS (*n* = 153), Web of Sciences (*n* = 145), and SPORTDiscus (*n* = 10)], from which 305 duplicates were removed, resulting in 221 unique records. Following title and abstract screening, 17 articles were excluded because they did not meet inclusion criterion six. The full texts of the remaining 204 articles were reviewed, and 41, 146, and 1 article were excluded on the basis of exclusion criteria one, two, and four, respectively. Consequently, 16 articles fulfilled all the inclusion criteria and were incorporated into the final qualitative synthesis (see Figure 1).

### 3.2 Quality assessment

Quality assessment was conducted by two independent authors via design-appropriate tools: RoB 1 for RCTs (*n* = 7), ROBINS-I for nonrandomized studies (*n* = 6), and JBI checklists for retrospective studies (*n* = 2) and case series (*n* = 1). Overall, the methodological quality was moderate, with 68.7% of the studies showing a moderate risk of bias, 12.5% a serious risk, and 25% an unclear risk. Selection bias was the most problematic domain, particularly in nonrandomized studies, where 66.7% of the studies presented serious risk due to self-selection of participants into voluntary before-school programs. Performance bias was universally high (100%) across all intervention studies because of the inherent inability to blind participants to PA interventions. Among RCTs, quality varied substantially: Garcia-Hermoso et al. (54) demonstrated the strongest methodological rigor, whereas Smith et al. (47) reported high risk across multiple domains. Nonrandomized studies generally showed a moderate risk of bias, with serious issues in participant selection for several studies. Cradock et al. (48) reported the lowest overall risk. In detail, the quality assessment for this systematic review can be found in Supplementary Data Sheet 1.

### 3.3 Characteristics of the included studies

The characteristics of the included studies can be found in Table 1.

## 4 Discussion

This study aims to systematically analyze the effects of before-school PA programs to highlight strategies that can be implemented to improve children's health and cognitive development. The main findings reveal that before-school exercise programs (BESP) and school-based interventions that integrate PA before school hours induce significant changes in cognitive processes, attention capacity, academic success, improvement in school climate, cardiorespiratory performance, and prevention of childhood obesity. To date, several programs have been published, and their effects have been evaluated when preschool PA interventions are incorporated. For this reason, this systematic review aims to synthesize the characteristics of these programs and the reported results to highlight novel strategies to improve children's health and development.

In the scientific literature, there is considerable interest in understanding the effects of PA programs implemented before the start of the school day. The objective is to determine whether these programs improve PF, body composition, academic performance, and cognitive function, among other factors (15, 59). Some studies have shown that movement-based games, which are conducted 3 times a week over 8 weeks for overweight children aged 10–12 years, can enhance inhibitory control (F (1, 84) = 22.25, *p* < 0.001, partial η^2^ = 0.21) (59). Another study by Schmidt et al. (60) evaluated executive functions through a 6-week intervention, twice weekly. Experimental group 1 participants in games with high cognitive involvement; group 2 participants in aerobic exercises with reduced cognitive demands; and the control group participants engaged in activities with low PF and cognitive effort. All the groups improved in terms of the inhibition variable (χ^2^(1, *N* = 181) = 4.50, *p* < 0.05), but there were no differences between the groups (*p* = 0.94). These findings imply that intervention effects can depend on measurement intervals, sample characteristics, or the intensity of the intervention. Thus, additional diverse studies across various contexts that use standardized variables and instruments are necessary.

In this context, some studies have examined the relationships among PA, participation in physical education programs, and school sports with academic performance and have concluded that incorporating PA into the school curriculum is necessary to improve academic performance. A study conducted by Fritz et al. (61) revealed that 9 years of schooling from ages 6–8, with 40 min per week, improves academic performance. Other studies have shown that higher levels of PA are associated with better cognitive performance when PA increases from 60 to 200 min per week (62). It appears that the amount of PA per week plays a key role in influencing academic and cognitive performance variables. However, it remains to be determined whether this time is related to the intensity of the PA performed, which would require further research in the school context.

These results show that incorporating PE programs does not always yield the same outcomes, as they depend on when they are implemented and, especially, on the domains evaluated and researched. This opens the door for future studies investigating the effects of different PA programs before school at different ages in preschool and school. A systematic review of randomized controlled trials on cognitive competence revealed that PE promotes cognitive abilities (creativity, attention, memory), inhibitory control, and academic performance (15). However, there is concern about students' health when the time devoted to physical education programs is reduced without affecting academic success (63). Given the above, there is a need to understand how different variables of PA, cognitive parameters, academic performance, health, PF, and school climate interact.

### 4.1 Effects of preschool-aged PA interventions on cognitive parameters and academic performance

Participation in physical activities improves learning and certain cognitive and academic areas in children. However, the main difficulty in establishing the influence at school age lies in the methodological diversity of the studies, with some reporting positive associations between PA and cognitive performance, whereas others reporting no associations (64). Different programs, such as MiM-Maze and yoga, which last 30 min a day and 5 times a week, can improve students' academic skills (50). Similarly, the Active-Start intervention caused changes in children's academic performance, especially in mathematics (0.63, 95% CI: 0.49–0.77) and language (0.49, 95% CI: 0.32–0.66), with significant differences (*p* < 0.001) after an 8-week program lasting approximately 30 min before the start of the first class. The study conducted by Korcz et al. (58) revealed that auditory and linguistic functions are stimulated in children aged 8–9 years when they attend PE classes 3 times a week for 15 min over 12 weeks before the first school bell rings, with a corresponding improvement in cognitive activity (*p* < 0.01). This diversity in results is due not only to the age at which it is assessed but also to the study design; the domains evaluated in academic performance; the duration, frequency, and type of intervention; and the instruments (objective/subjective) used.

Conversely, a systematic review of the influence of PA and PF on the academic achievements of school-age children revealed that the heterogeneity of studies makes it difficult to determine whether this influence depends on the PA dose (65). These disparities in the scientific literature stem from the diversity of methodological criteria used to assess PA, the context in which it is analyzed, the variables considered, and the instruments used to assess its effects on cognitive parameters. This presupposes an opportunity to recognize the didactic potential of incorporating school-based PA interventions in the school environment (45, 46).

### 4.2 Effects of preschool PA interventions on health parameters and physical fitness

A study conducted with kindergarten children in the United States, in which the daily step count and minutes of moderate-to-vigorous PA (MVPA) were evaluated, revealed that children spent ~16.3 min in MVPA during sessions and that 33% of the session time was MVPA; however, MVPA appeared to have a limited effect on self-reported quality of life and BMI (44). On the other hand, scientific evidence has shown that interventions developed to promote PA before school hours have positive effects on MVPA performance (>15%) (66). The evidence is consistent in that preschool interventions significantly increase MVPA time and improve PF in the short term, with a possible benefit in combating obesity and preventing the acquisition of sedentary behaviors from an early age (67). This study also revealed that the greatest difference was in relation to sex, with a significant difference for girls (6.8 vs. 6.1 min/h of MVPA) but not for boys (7.9 vs. 7.2 min/h of MVPA) (68).

Likewise, it has been reported in other studies that the incorporation of before-school PA interventions could favor children's willingness to maintain greater adherence to regular exercise without causing a decrease in movement during the school day and without academic implications (44, 52). The evidence is consistent in that before-school interventions considerably increase MVPA time and improve physical fitness in the short term, with a possible benefit to combat obesity and the acquisition of sedentary behaviors from an early age (68). Another meta-analytic study concluded that aerobic exercise produces significant changes in variables such as general executive function (SMD = −0.50; 95% CI [−0.68, −0.32]; *p* < 0.01), working memory (SMD = −0.63; 95% CI [−1.06, −0.20]; *p* < 0.01), and inhibitory control (SMD = −0.52; 95% CI [−0.72, −0.31]; *p* < 0.01), although it does not produce significant differences in the cognitive flexibility of overweight and obese children (SMD = −0.32; 95% CI [−0.71, 0.07]; *p* = 0.11) (64). In contrast, some domains of PF and health, especially aerobic exercise, have been reported to be associated with cognitive factors in schoolchildren. Therefore, research should continue to examine the effects of different programs that relate physical variables to cognitive indicators to determine whether the same intervention protocols produce the same changes across contexts, ages, and domains of PF.

### 4.3 PA and school climate

A study conducted by Hormazábal-Aguayo et al. (52), which sought to determine the influence of PA on bullying in students with social vulnerability, revealed that a PA program significantly affected the probability of suffering physical bullying (OR = 0.18; 95% CI 0.04–0.82; *p* = 0.027) and verbal bullying (OR = 0.13; 95% CI 0.02–0.97; *p* = 0.046). In that same sense, it is necessary to understand the social determinants and the relationships they have with academic performance and students' health, which are sociodemographic factors that induce school-based PA intervention programs to regulate the vulnerabilities of certain less favored contexts (65). This is important because it has been reported that socioeconomic factors are related to infants' health and development, and, in another way, there may be barriers or possibilities for educational centers to favor school-based PA interventions according to the possibilities offered by each context (66). Similarly, another study that investigated the effects of PA at school on attention, academic performance, and relationships with students who have intellectual disabilities revealed that the incorporation of PA serves as a strategy to promote socialization and, in turn, produces improvements in academic performance and attention (69).

### 4.4 Duration, frequency, and intensity of intervention programs

The study conducted by Cradock et al. (48), which investigated the effects of a PA program before school called BOKS (Build Our Kids' Success) over 12 weeks with weekly evaluation per school, a frequency of 2–3 days per week, and an average intervention duration of 47 min with 33 min of observed PA time, revealed that students accumulated more PA, including total steps (1503, 95% CI: 1286–1719, *p* < 0.001), minutes of MVPA (13.4, 95% CI: 11.3–15.5, *p* < 0.001), minutes of vigorous PA (VPA) (4.5, 95% CI: 3.6–5.5, *p* < 0.001), and minutes of total PA (TPA) (24.1, 95% CI: 17.3–30.9, *p* < 0.001).

Similarly, another study that used the BOKS program with 1,229 children between 5 and 14 years of age from 24 schools, where 16 schools administered the program 2 days per week and 8 schools 3 days per week, reported that the 3-day-per-week program resulted in a significant change in the adjusted mean BMI z score (0.51 ± 0.14 at baseline and 0.29 ± 0.14 at 12 weeks), higher odds of being in a lower BMI category (OR = 1.35; 95% CI = 1.12; 1.62), and greater student engagement (difference of 0.79 units, 95% CI = −0.01, 1.60), positive affect (difference of 1.41 units, 95% CI = 0.16, 2.65), and vitality/energy compared with the 2-times-per-week group (difference of 0.60 units, 95% CI = 0.11, 1.08) (53). Another “Active-Start” intervention with 170 children between 8 and 10 years of age from three schools with a duration of 30 min over 8 weeks resulted in a decrease in blood pressure at the beginning of the intervention (*p* < 0.01), although it was not different from that of the control group, and the effect of PA on blood pressure tended to disappear in children exposed to excessive sedentary time (49).

A study that investigated the relationship of PA with positive health outcomes in 27 children between 8 and 10 years of age measured the level of PA in relation to different exercise intensities (moderate intensity with HR between 25% and 40% of heart rate reserve, vigorous intensity > 40% of heart rate reserve) 3 days per week over an 8-week period, concluding that the Department of Health activity goals were achieved and that the intervention group (*n* = 11) developed 18 more minutes of MVPA (55). Another study that sought to incorporate a PA program with aerobic exercises between 15 and 20 min allowed students to be active 1 h and 40 min more per week (68). It is well known that PA not only has effects on reducing sedentary behaviors but also provides benefits at the mental and cognitive levels. In this context, the study conducted by Willis et al. (57) revealed that 15–20 min of cooperative PA for 8 weeks and 3 times per week improved behavior, PA levels, and adherence to remaining active.

Finally, the study conducted by Korcz et al. (58) sought to understand the effects of two school interventions (PAnvs. cognitive engagement) on academic skills in 88 children aged 8–9 years. The intervention lasted 12 weeks with 3 weekly 15-minute sessions before school, indicating that physical activities, when combined with cognitive/computer interventions, prove to be more effective than cognitive activities alone when seeking to improve academic skills (58). This finding reinforces the value of PA for incorporation into different school contexts in the period before entering school, with the goal of improving PA levels, core executive functions, academic performance, etc. (70). Other programs that have been conducted have sought to analyze the effects of short relaxation training with mindfulness and its influence on executive functions and cortisol levels. Fifty-one children who participated in a between-subjects pilot study and who completed 5 sessions of 30 min of relaxation training with mindfulness showed a difference in their cortisol levels (*p* = 0.026, η^2^ = 0.134), although there were no effects on executive function skills.

## 5 Limitations of the study

Importantly, the present review has several limitations that must be considered. This review is based on heterogeneous data (cross-sectional designs) with diverse methodological qualities, making it difficult to establish causal relationships between the implementation of school-based PA interventions and the effects that can be produced at the cognitive, physical, and social levels. Specific characteristics of methodological quality, such as the blinding of participants, evaluators, or researchers, are common in studies with interventions in educational settings for schoolchildren.

Another limitation is associated with the diversity in students' age and the quantification of PAs performed, which makes it difficult to understand the effects on physical fitness, cognitive function, and academic performance in children, making it difficult to perform a meta-analysis. Finally, the diversity in the types of intervention programs, evaluated variables, and instruments used makes it difficult to generalize the type of PA and exercise intensity necessary to induce significant changes in different variables related to other domains of the school environment.

Despite these methodological constraints, the evidence supports the integration of PA within educational settings as a public health strategy, with physical education serving as the primary institutional mechanism for systematic PA delivery in preschool and school contexts.

Additional high-quality studies, such as randomized controlled trials, longitudinal studies, and multicenter studies, are needed to determine which specific doses of PA have an impact on the response to age and each specific context.

## 6 Conclusion

PA intervention before class can improve academic performance and physical fitness in schoolchildren, although its effects on other cognitive domains, such as attention and concentration; physical characteristics, such as BMI; and social aspects, such as quality of life and physical and verbal school bullying, depend on how much physical improvement children achieve.

School-based interventions that consider PA performance can constitute a setting that allows children to be more willing to learn and achieve better use of school-free time to improve cardiorespiratory fitness and prevent an increase in childhood obesity. Translating these findings into practice requires educational institutions to prioritize evidence-based curricula design, ensure adequate time allocation for PA throughout the school week, and establish quality standards for program implementation and monitoring.

## Data Availability

The original contributions presented in the study are included in the article/Supplementary material, further inquiries can be directed to the corresponding author.
