# Supplementary material for: Effects of before-school physical activity programs on preschool and school children: a systematic review
Source: Front Public Health. 2026 Jul 28;14:1784551. doi: 10.3389/fpubh.2026.1784551 (PMC13457486; doi:10.3389/fpubh.2026.1784551)
Supplement: Supplementary file 1 [file Data_Sheet_1.docx]

**QUALITY ASSESSMENT**

**Supplementary material 1.1**. *Characteristics of included studies*

| Reference | Study design | Sample | Before-school program | Test / Instruments | Variables | Results | Conclusions |
| --- | --- | --- | --- | --- | --- | --- | --- |
| García-Hermoso et al. [45] | Randomized controlled trial | Chile; n = 170 children (8–10 years old; ~43% girls) from 3 public schools, low SES | Active-Start, 8 weeks, 5 days/week, 30 min/session (cooperative physical games, dance, playground activities, MVPA intensity) delivered at 8:00–8:30 am before classes | d2 Test of Attention (paper-pencil); Official school grades in language & math; ALPHA test battery: JAMAR dynamometer (Hydraulic Hand Dynamometer® Model PC-5030 J1), standing long jump, 4×10m shuttle-run, 20m shuttle-run (VO₂max by Léger equation); TANITA SC-331 bioelectrical impedance analyzer; Seca 769 scale & Seca 220 stadiometer for BMI; waist circumference tape; GENEActiv accelerometer | Selective attention, concentration, language, math, BMI, waist circumference, fat mass, fat-free mass, muscular fitness, motor ability, CRF | Improvements in language and math performance. Reduction in fat mass and increase in fat-free mass. | An 8-week before-school PA program improved academic performance, body composition, muscular and cardiorespiratory fitness. CRF moderated effects on attention and concentration; benefits were evident only in children with greater CRF gains |
| García-Hermoso et al. [44] | Randomized controlled trial | Chile, 170 children, 10.5 ± 0.5 y, 52% boys | Active-Start; 8 weeks, 5 days/week, 30 min; MVPA; games, circuits, motor activities | Bullying Victimization Scale, anthropometrics | Bullying victimization (physical, verbal, relational), fitness | Reduced physical/verbal victimization significantly compared to control | Before-school PA can reduce bullying in disadvantaged children. |
| Terson De Paleville & Immekus [46] | Randomized controlled trial | USA, 60 elementary students, 8–9 y | Minds in Motion & Yoga; 6 weeks, 3x/week, 25 min | BOT-2, Woodcock-Johnson Tests | Motor proficiency, reading, math | Improved motor skills and academic measures in intervention groups | Short PA/yoga before school enhances motor and academic skills. |
| Hoza et al. [47] | Randomized controlled trial | USA, 202 children, mean age 6.8 y | Aerobic activity vs. sedentary; 12 weeks, 31 min/day | DBD Rating Scale, teacher reports | ADHD symptoms, emotion, peer functioning | Reduced inattention and mood problems; improved behavior | Before-school PA reduces ADHD-related symptoms. |
| Kulp & Zhu [48] | Retrospective case-control | USA, 84 children, grades 4–5 | 10 weeks, 1x/week, 45 min aerobic program | Fitness tests, reading/math assessments | Fitness, academic performance | Improved fitness and reading scores; no effect on math | Weekly before-school PA benefits fitness and reading. |
| Stylianou et al. [49] | Cross design study | USA, 88 students, grades 3–4 | Running/Walking Club; 8 weeks, 3x/week, 20 min | Accelerometers | Steps, MVPA | Significant increase in steps and MVPA; no compensatory decrease | Before-school walking/running clubs effectively increase PA. |
| Goh et al. [50] | Quasi-experimental | USA, 138 children, mean 10 y | 6 weeks, 3x/week, SEL-based PA | DESSA-Mini | Social-emotional learning | Improved SEL competencies | PA integrated with SEL before school supports wellbeing. |
| Hormazábal-Aguayo et al. [51] | Randomized controlled trial | Chile, 170 children, 10.5 y | Active-Start; 8 weeks, 5 days/week, 30 min | Bullying Victimization Scale | Bullying victimization | Significant reduction in bullying victimization | Structured PA reduces school bullying. |
| Cradock et al. [42] | Quasi experimental | USA, 426 students, grades K–6 | BOKS; 12 weeks, 3x/week, 40 min | Accelerometers | Steps, MVPA, VPA | Higher PA on program days | BOKS increases children’s PA before school. |
| Whooten et al. [52] | Quasi-experimental | USA, 707 students, 24 schools | BOKS; 12 weeks, 2–3x/week | BMI, wellbeing surveys | BMI z-score, socioemotional wellbeing | BMI z-score improved (3x/week group), wellbeing increased | Frequent BOKS sessions improve health indicators. |
| Brackney et al. [53] | Quasi-experimental | USA, 200 children | Dance-based play sessions | Heart rate, PA logs | PA intensity, enjoyment | Children engaged in MVPA; enjoyed activities | Dance-based PA feasible to promote MVPA. |
| Tompkins et al. [54] | Retrospective case-control | USA, 42 students | Self-selected PA sessions | Heart rate monitors, RPE | HR, perceived exertion | Obese children had higher HR and RPE vs. normal weight | Self-paced PA feasible; differences by weight status. |
| Willis et al. [55] | Quasi-experimental | USA, 88 students, 4th–5th grade | 8 weeks, 3x/week, 15–20 min; cooperative PA, TPSR model | Steps, Stroop test, academic progress | On/off-task behavior, PA, academics | Increased PA, SEL; no significant academic improvement | Before-school RTI improves behavior and PA. |
| Smith et al. [43] | Pilot intervention (solo un grupo) | USA, 17 children (K–3), ADHD risk | 26 min/day, 8 weeks, MVPA in stations | DBD, BOT-2, Shape School, teacher/parent ratings | ADHD symptoms, motor, cognition, behavior | Improvements in inhibition, behavior, ADHD symptoms | Before-school PA promising for ADHD symptom reduction. |
| Lyster et al. [56] | Randomized trial | Norway, 269 children, preschool, 5–6 y | Morphological vs. phonological awareness; 17 weeks, 30 min/week | Reading tests (grade 1 & 6) | Reading, comprehension | Morphological training improved long-term reading comprehension | Preschool morphological training enhances literacy long-term. |
| Koncz et al. [57] | Pilot Randomized Controlled Trial | Hungary, 51 preschoolers (mean 6.8 y) | Mindfulness-relaxation, 1 week, 5x 30 min | Digit span, Go/No-Go, cortisol sampling | Executive functions, cortisol | No EF effect; boys showed reduced cortisol post-school entry | Short mindfulness feasible; stress-buffer in boys. |
| Korcz et al. [58] | Randomized Controlled Trial | Poland, 88 children, 8–9 y | Active Before School Bell; 12 weeks, 3x/week, 15 min (PA vs cognitive) | Eurofit, academic skills battery, PA questionnaires | Academic skills, fitness, PA attitudes | PA group improved visual-spatial; both experimental > control in language skills | PA before school effective for academic and fitness outcomes. |

DBD = Disruptive Behavior Disorders Rating Scale
BOT-2 = Bruininks–Oseretsky Test of Motor Proficiency, 2nd ed.
WRAML-2 = Wide Range Assessment of Memory and Learning, 2nd ed.
ActiGraph GT3X = Tri-axial accelerometer for PA assessment
PACER = Progressive Aerobic Cardiovascular Endurance Run (FitnessGram)
MVPA = Moderate-to-vigorous physical activity
VPA = Vigorous physical activity
RTI = Response to Intervention
SEL = Social and Emotional Learning
HBSC = Health Behaviour in School-aged Children survey
APAS = Attitudes toward PAScale

**Supplementary material 1.2.** *Risk of bias in non-randomized studies*

| Study | Bias due to confounding | Bias in selection of participants into the study | Bias in classification of interventions | Bias due to deviations from the intended intervention | Bias due to missing data | Bias in measurement of outcomes | Bias in selection of the reported result |
| --- | --- | --- | --- | --- | --- | --- | --- |
| Willis et al., [55] | Moderate | Serious | Moderate | Moderate | Moderate | Moderate | No information |
| Goh et al., [50] | Serious | Serious | Low | Moderate | Moderate | Moderate | No information |
| Cradock et al., [42] | Low | Serious | Low | Moderate | Moderate | Low | Low |
| Stylianou et al., [49] | Moderate | Moderate | Low | Moderate | Moderate | Moderate | No information |
| Hormazabal-Aguayo et al., [51] | Moderate | Serious | Moderate | Moderate | Moderate | Moderate | Low |
| Whooten et al., [52] | Moderate | Moderate | Low | Moderate | Moderate | Moderate | Low |
| Stylianou et al. [49] | Serious | Serious | Moderate | Moderate | Low | Low | No information |

**Supplementary Material 2.3.** *Risk of bias in randomized trials*

| Study | Sequence Generation | Allocation Concealment | Blinding Participants/Personnel | Blinding Outcome Assessor | Incomplete Data | Selective Reporting | Other Bias |
| --- | --- | --- | --- | --- | --- | --- | --- |
| Garcia-Hermoso et al. [45] |  |  |  |  |  |  |  |
| Korcz et al. [58] |  |  |  |  |  |  |  |
| Smith et al. [43] |  |  |  |  |  |  |  |
| Lyster et al. [56] |  |  |  |  |  |  |  |
| Koncz et al. [57] |  |  |  |  |  |  |  |
| Garcia-Hermoso et al. [44] |  |  |  |  |  |  |  |
| Terson De Paleville & Immekus [46] |  |  |  |  |  |  |  |

|  | Low risk of bias |
| --- | --- |
|  | Unclear risk of bias |
|  | High risk of bias |

**Supplementary Material 1.4.** JBI critical appraisal checklist for case control studies

| **Study** | **D1** | | **D2** | | **D3** | **D4** | **D5** | **D6** | **D7** | **D8** | **D9** | **D10** | **Overall Risk of Bias** |  |
| --- | --- | --- | --- | --- | --- | --- | --- | --- | --- | --- | --- | --- | --- | --- |
| Kulp & Zhu [48] |  | | N/A | | N/A |  |  |  |  |  |  |  |  |  |
| Tompkins et al., [54] |  | | N/A | | N/A |  |  |  |  |  |  |  |  |  |
|  | |  | | “Yes” for domains/ Low risk for overall risk of bias | | | | | | | | | | |
|  | |  | | “Unclear” for domains/ Moderate for overall risk of bias | | | | | | | | | | |
|  | |  | | “No” for domains/ High risk for overall risk of bias | | | | | | | | | | |

D1:Were the groups comparable other than the presence of disease in cases or the absence of disease in controls?; D2:Were cases and controls matched appropriately?; D3: Were the same criteria used for identification of cases and controls?;D4: Was exposure measured in a standard, valid and reliable way?; D5: Was exposure measured in the same way for cases and controls?; D6: Were confounding factors identified?; D7: Were strategies to deal with confounding factors stated?; D8: Were outcomes assessed in a standard, valid and reliable way for cases and controls?; D9: Was the exposure period of interest long enough to be meaningful?; D10: Was appropriate statistical analysis used?

**Supplementary Material 1.5.** JBI Critical Appraisal Checklist for Case Series

| **Study** | **D1** | | **D2** | | **D3** | **D4** | **D5** | **D6** | **D7** | **D8** | **D9** | **D10** | **Overall Risk of Bias** |  |
| --- | --- | --- | --- | --- | --- | --- | --- | --- | --- | --- | --- | --- | --- | --- |
| Smith et al., [43] |  | |  | |  |  |  |  |  |  |  |  |  |  |
|  | |  | | “Yes” for domains/ Low risk for overall risk of bias | | | | | | | | | | |
|  | |  | | “Unclear” for domains/ Moderate for overall risk of bias | | | | | | | | | | |
|  | |  | | “No” for domains/ High risk for overall risk of bias | | | | | | | | | | |

D1: Were there clear criteria for inclusion in the case series?; D2: Was the condition measured in a standard, reliable way for all participants included in the case series?; D3: Were valid methods used for identification of the condition for all participants included in the case series?; D4: Did the case series have consecutive inclusion of participants?; D5: Did the case series have complete inclusion of participants?; D6: Was there clear reporting of the demographics of the participants in the study?; D7: Was there clear reporting of clinical information of the participants?; D8: Were the outcomes or follow up results of cases clearly reported?; D9: Was there clear reporting of the presenting site(s)/clinic(s) demographic information?; D10: Was statistical analysis appropriate?
